# Soluble RANKL expression in *Lactococcus lactis* and investigation of its potential as an oral vaccine adjuvant

**DOI:** 10.1186/s12865-015-0132-x

**Published:** 2015-11-25

**Authors:** Jeong-In Kim, Tae-Eun Park, Sushila Maharjan, Hui-Shan Li, Ho-Bin Lee, In-Seon Kim, Dachuan Piao, Jun-Yeong Lee, Chong-Su Cho, Jin-Duck Bok, Zhong-Shan Hong, Sang-Kee Kang, Yun-Jaie Choi

**Affiliations:** Department of Agricultural Biotechnology & Research Institute for Agriculture and Life Sciences, Seoul National University, Seoul, South Korea; Institute of Green-Bio Science & Technology, Seoul National University, Pyeongchanggun, Gangwondo South Korea; Department of Animal Science, Tianjin Agricultural University, Tianjin, China

**Keywords:** Oral adjuvant, RANKL, M cells, *L. lactis*, Mucosal immunization

## Abstract

**Background:**

To initiate mucosal immune responses, antigens in the intestinal lumen must be transported into gut-associated lymphoid tissue through M cells. Recently, it has been increasingly recognized that receptor activator of NF-kB ligand (RANKL) controls M cell differentiation by interacting with RANK expressed on the sub-epithelium of Peyer’s patches. In this study, we increased the number of M cells using soluble RANKL (sRANKL) as a potent mucosal adjuvant.

**Results:**

For efficient oral delivery of sRANKL, we constructed recombinant *Lactococcus lactis* (*L. lactis*) IL1403 secreting sRANKL (sRANKL-LAB). The biological activity of recombinant sRANKL was confirmed by observing RANK-RANKL signaling in vitro. M cell development in response to oral administration of recombinant *L. lactis* was determined by 1.51-fold higher immunohistochemical expression of M cell marker GP-2, compared to that of non-treatment group. In addition, an adjuvant effect of sRANKL was examined by immunization of mice with M-BmpB as a model antigen after treatment with sRANKL-LAB. Compared with the wild-type *L. lactis* group, the sRANKL-LAB group showed significantly increased systemic and mucosal immune responses specific to M-BmpB.

**Conclusions:**

Our results show that the M cell development by sRANKL-LAB can increase the antigen transcytotic capability of follicle-associated epithelium, and thereby enhance the mucosal immune response, which implies that oral administration of sRANKL is a promising adjuvant strategy for efficient oral vaccination.

**Electronic supplementary material:**

The online version of this article (doi:10.1186/s12865-015-0132-x) contains supplementary material, which is available to authorized users.

## Background

The primary importance of oral vaccination is that many infections start from the gastrointestinal tract, and in these cases, topical application of a vaccine is usually required to induce a gut mucosal immune response [1]. However, oral vaccination often does not induce strong IgA responses because the majority of antigens cannot reach the mucosal inductive sites in the harsh digestive conditions of the gastrointestinal tract. Further, ingested antigens can induce a state of immune tolerance [2]. Given the poor immunogenicity of oral vaccines, the use of an appropriate mucosal adjuvant may be important for successful mucosal vaccination. Although some toxin adjuvants including cholera toxin and *E. coli* heat-labile toxin have been suggested as mucosal vaccine adjuvants, safety concerns prevent their use in clinical applications. Several cytokines including interleukin-6 [3], −12 [4], −15 [5] and Type I interferon-γ [6] have also been investigated as safe and non-toxic mucosal adjuvants; however, they have generally showed poor efficacy [3–6]. Hence, we need a new approach to enhance mucosal immunity in response to oral vaccines.

M cells are specialized epithelial cells in the follicle-associated epithelium (FAE) that overlies gut-associated lymphoid tissue (GALT) in Peyer’s patches. M cells transport luminal organisms and particles passing through the intestine toward the GALT, and thus play a central role in the initiation of an intestinal immune response [7]. M cells account for only 10 % of FAE cells in rodents, and 5 % in humans [8]. Due to the low numbers of M cells in the intestinal tract, targeting M cells using synthetic peptides [9] or pathogen-exploited molecules [10, 11] could be a promising approach for enhancing oral vaccine potency.

D*e novo* differentiation of M cells is stimulated by pathogens or foreign antigens, and induces up-regulation of transport in Peyer’s patches, thereby enhancing protective immune responses [12, 13]. Increasing the number of M cells can thus be a promising biomimetic strategy to enhance the efficacy of an oral vaccination. In recent, the importance of receptor activator of NF-kB ligand (RANKL) in controlling M cell differentiation in Peyer’s patches has been increasingly recognized [14–16]. RANKL is a member of the tumor necrosis factor superfamily that has diverse functions mediated by its interaction with RANK. In the body, RANKL is produced as a transmembrane protein, but it can be cleaved by several metalloproteinases [17, 18] and released in its soluble extracellular form (sRANKL). RANK-RANKL molecular signaling is an essential regulator of bone remodeling, inducing the fusion of osteoclast progenitors into osteoclasts [19], and important in the establishment of the thymic microenvironment and the lymph node [20]. In Peyer’s patches, RANKL expression by subepithelial stromal cells shows a polarized pattern, while RANK is expressed throughout the epithelial cells of the small intestine [21]. This localization indicates a possible function of RANKL in gut mucosal immunity. The role of RANKL in M cell development was first demonstrated in vivo by the finding that RANKL null mice have less than 2 % of wild-type levels of M cells, and the number of M cells is rescued by administration of exogenous RANKL for 7 days [15]. RANKL induces the expression of the Ets transcription factor Spi-B in epithelial precursors, which differentiate into M cells [16].

Here, we examined the adjuvant potential of RANKL, expecting that oral delivery of recombinant RANKL would increase the number of M cells in the intestine. For efficient oral delivery of sRANKL, *Lactococcus lactis* (*L. lactis*) IL1403 was used as a live carrier because it protects the recombinant protein from the low pH of the stomach, and from enzymatic degradation during passage through the intestinal tract. [22, 23]. The biological activity of sRANKLs was confirmed by observing RANK-RANKL signaling in vitro. M cell development in response to oral recombinant *L. lactis* was demonstrated by staining with GP-2, an M cell marker. sRANKL enhanced the protective antibody response against a model subunit antigen, M-BmpB (*Brachyspira* membrane protein B conjugated with CKS9) [24] developed to protect pigs from Brachyspira hydrosenteriae, which causes muco-hemorrhagic dysentery [25].

## Results

### Production and secretion of sRANKLs from recombinant *L. lactis*

**Fig. 1:**
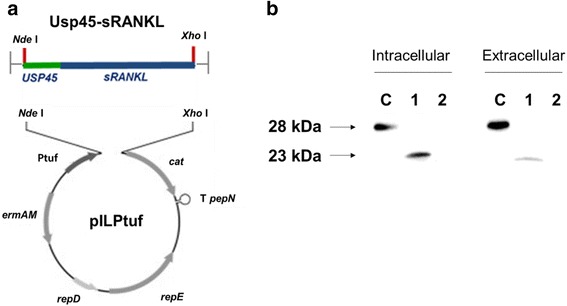
**a** Schematic diagram for construction of recombinant sRANKL expression vector system (modified from [3]). **b** Western blot for detecting sRANKL from cell extracts (intracellular) and concentrated culture supernatants (extracellular). C: commercial sRANKL; lane 1: sRANKL-LAB; lane 2: WT-LAB

The recombinant *L. lactis* IL 1403 expressing secretory form of 181 amino acid sRANKL [15] (sRANKL-LAB) was prepared using pILPtuf vector system previously constructed by our group [26]. To permit the production and secretion of protein, sRANKL was conjugated with Usp45 signal peptide [3]. The schematic illustration of gene constructs and expression vector system is shown in Fig. 1a.

To examine the expression and secretion of sRANKLs from recombinant sRANKL-LAB, the cytosolic and secreted protein fractions were separately prepared and analyzed by Western blot. The bands of sRANKL protein from intracellular and extracellular (secreted) protein of *L. lactis* on Western blot are shown in Fig. 1b. The sRANKLs was detected in lane C (commercial sRANKL; ~28 kDa when glycosylated), lane 1 (cytosolic sRANKL from sRANKL-LAB; ~23 kDa) while no band was detected in lane 2 (wild-type *L. lactis* IL1403). In addition, band of sRANKL was detected from secreted protein fraction of sRANKL-LAB, confirming that conjugation of Usp45 signal peptide greatly improved secretion of sRANKL. The secreted protein had lower molecular weight than cytosolic sRANKL because Usp45 signal peptide was detached from protein while secreted from cells. For quantification of sRANKL expression level, enzyme-linked-immuno-sorbent assays (ELISA) was conducted. It was determined that 10^9^ CFU/ml (OD_600nm_ = 2; culture volume = 500 ml) of sRANKL-LAB produces 193 ng/ml of sRANKL and secrets 60 ng/ml.

### Physiological characterization of sRANKL-LAB

**Fig. 2:**
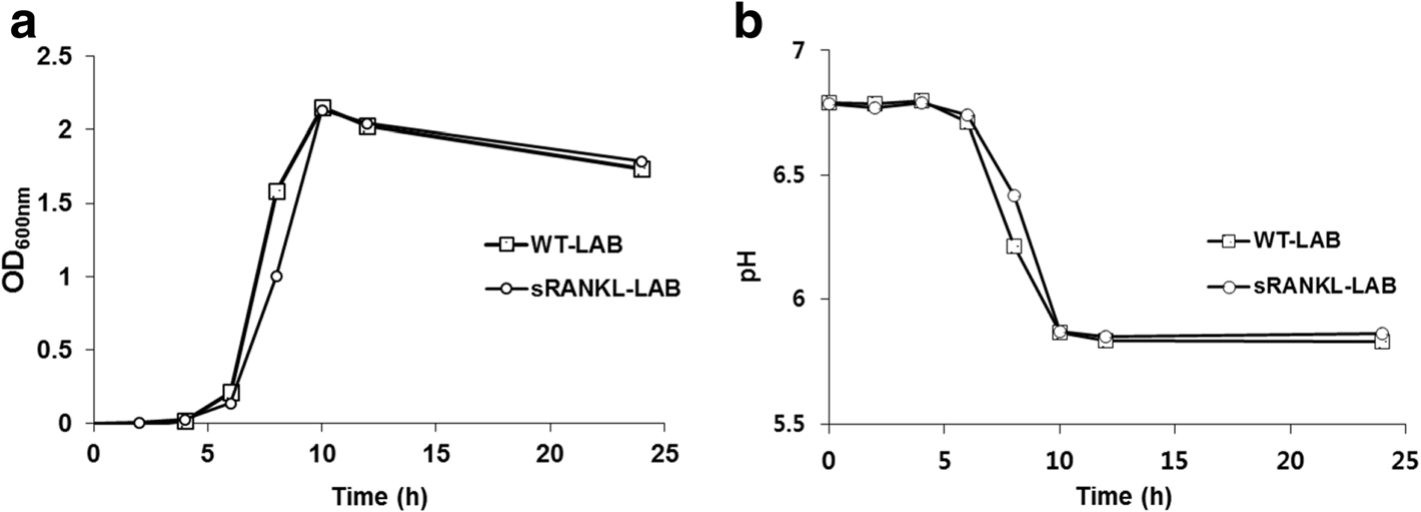
Physiological characterization of recombinant sRANKL-LAB. **a** Growth of WT-LAB and sRANKL-LAB was traced by measuring OD value at a wavelength of 600 nm. **b** The pH of WT-LAB and sRANKL-LAB was measured. The growth and pH were measured every 2 h for 12 h and at 24 h after inoculation

To examine the physiological characteristics of sRANKL-LAB under metabolic burden of foreign protein expression, patterns of growth and lactic acid production of sRANKL-LAB were analyzed during cultivation at 30 °C. As shown in Fig. 2 all of groups showed similar patterns of growth and pH decrease. Although sRANKL-LAB showed slightly delayed growth rate compared to wild-type *L. lactis* IL1403 (WT-LAB), there was no statistical difference (Fig. 2a), and all of groups reached stationary phase and minimum level of pH at 10 h of cultivation (Fig. 2b). These results suggest that production of sRANKL does not affect to normal physiology in sRANKL-LAB.

### Validation of the biological activity of recombinant sRANKLs in vitro

We tested the biological activity of recombinant sRANKL produced by sRANKL-LAB by observing the stimulation of RANK-RANKL signaling in vitro. RAW 264.7 cells (mouse leukemia monocyte macrophage) were used for the assay because they express endogenous RANK and are able to form osteoclast-like cells in response to RANKL [27]. We analyzed the expression of key molecules in the RANK-RANKL signaling pathway (TRAF6, NFATc1 and TRAP) that are related to osteoclast differentiation.

Culture media containing secreted sRANKL was directly applied to RAW264.7 cells at 20 ng/ml of recombinant sRANKL. In addition, media from WT-LAB was used as a negative control, and commercial sRANKL as a positive control [28].

**Fig. 3:**
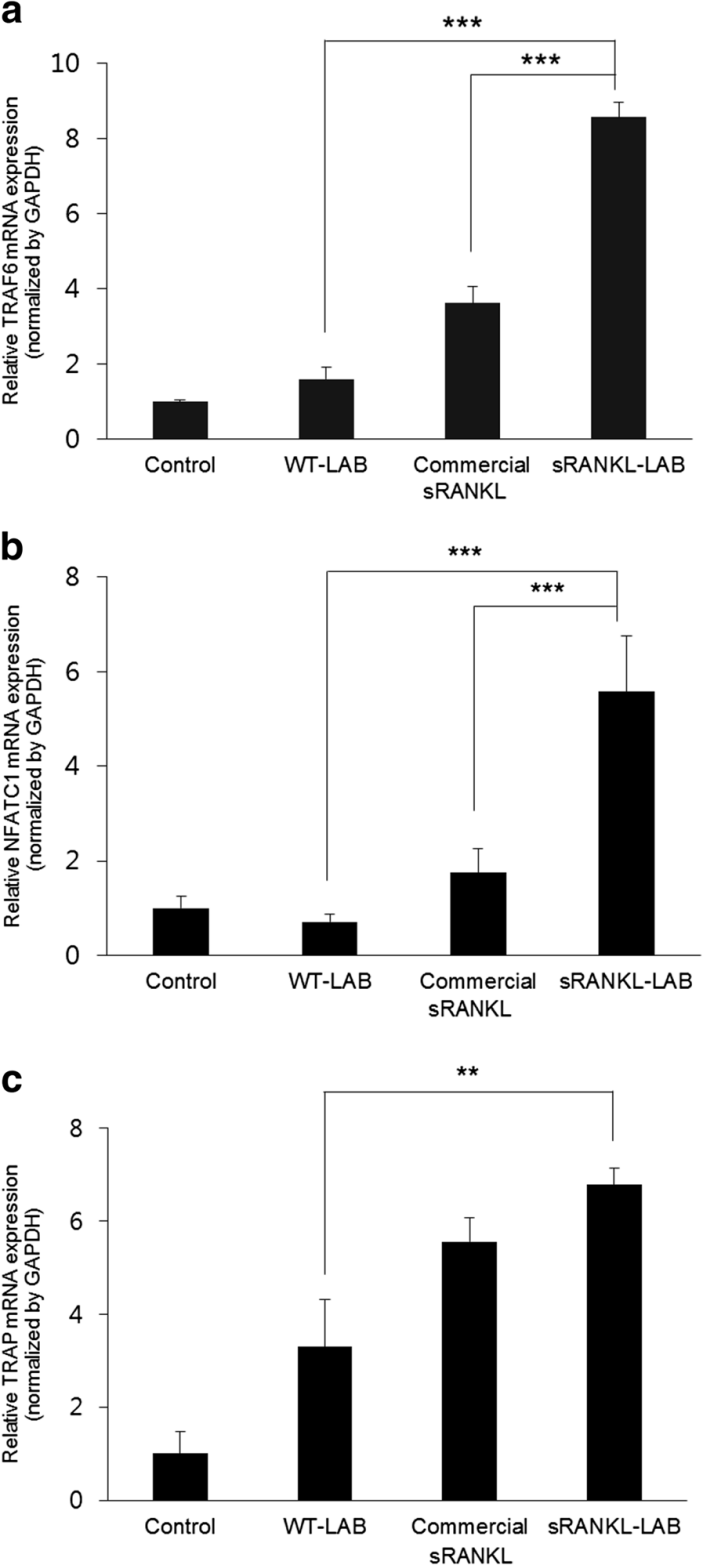
qRT-PCR analysis of RANK-RANKL signaling-related gene expression to validate the functional activity of sRANKL. The mRNA expressions of **a** TRAF6, **b** NFATc1 and **c** TRAP were analyzed at day 6 after exposure of media of WT-LAB, commercial sRANKL (20 ng/ml) or media of sRANKL-LAB containing 20 ng/ml of sRANKL to RAW 264.7 cells. The mRNA levels were normalized by GAPDH expression, and expressed as relative gene expression compared to control. For significance tests, a one-way analysis of variance (ANOVA) followed by Tukey’s post-hoc test were used, and expressed as follows; *P < 0.05, **P < 0.01, ***P < 0.001

To optimize the concentration of sRANKL, a variety of concentrations of commercial sRANKL was applied to RAW264.7 cells, and the expression of RANK-RANKL signaling molecules was analyzed. TRAF6, NFATc1 and TRAP mRNA expression levels were increased by increasing the concentration of purified commercial sRANKL protein. We used 20 ng/ml in our experiments because it sufficiently elicited RANK-RANKL signaling (Additional file 1: Figure S1). As shown in Fig. 3a-c, treatment with culture media containing recombinant sRANKL (20 ng/ml) also induced significant increases of TRAF6, NFATc1 and TRAP mRNA compared with the WT-LAB group, implying that recombinant sRANKL can elicit RANK-RANKL signaling transduction. The efficacy of induction of RANK-RANKL signaling molecules by recombinant sRANKL was higher than commercial RANKL at the same concentration, which may be due to the different origins of sRANKL and the different positions in sRANKL expression sites.

## M cell development induced by recombinant sRANKL in vivo

**Fig. 4:**
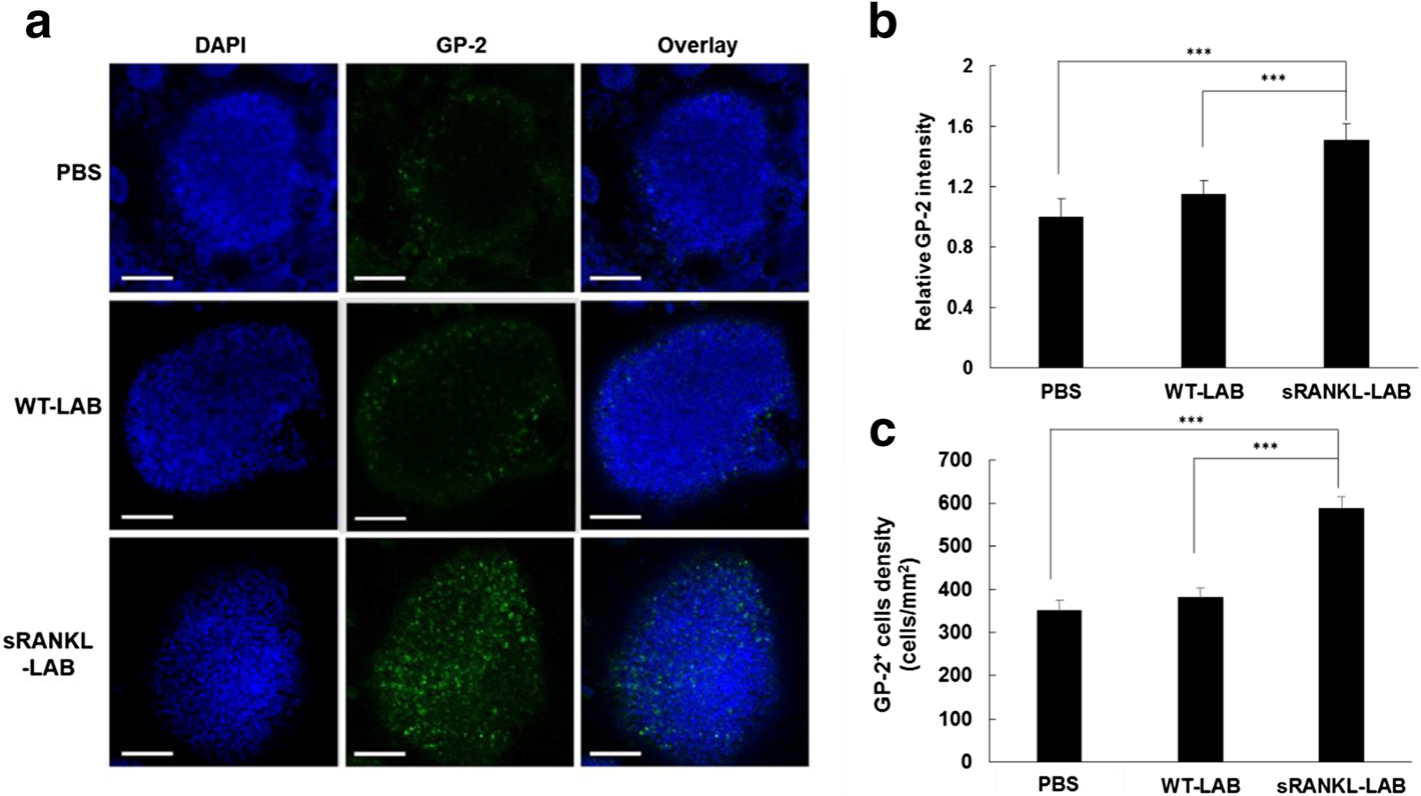
**a** Representative images for whole mount IHC of M cells in the FAE. At day 8 post-treatment of WT-LAB or sRANKL-LAB for consecutive 7 days, Peyer’s patches from mice of each group was isolated and stained with Alexa 488-labeled anti-GP-2, to observe M cell development. The nucleus was stained with DAPI. Scale bar represents 100 μm. **b** and **c** the GP-2^+^ intensity and the GP-2^+^ cell density in the FAE of each group were analyzed using ImageJ software. For significance tests, a one-way analysis of variance (ANOVA) followed by Tukey’s post-hoc test were used, and expressed as follows; *P < 0.05, **P < 0.01, ***P < 0.001

**Fig. 5:**
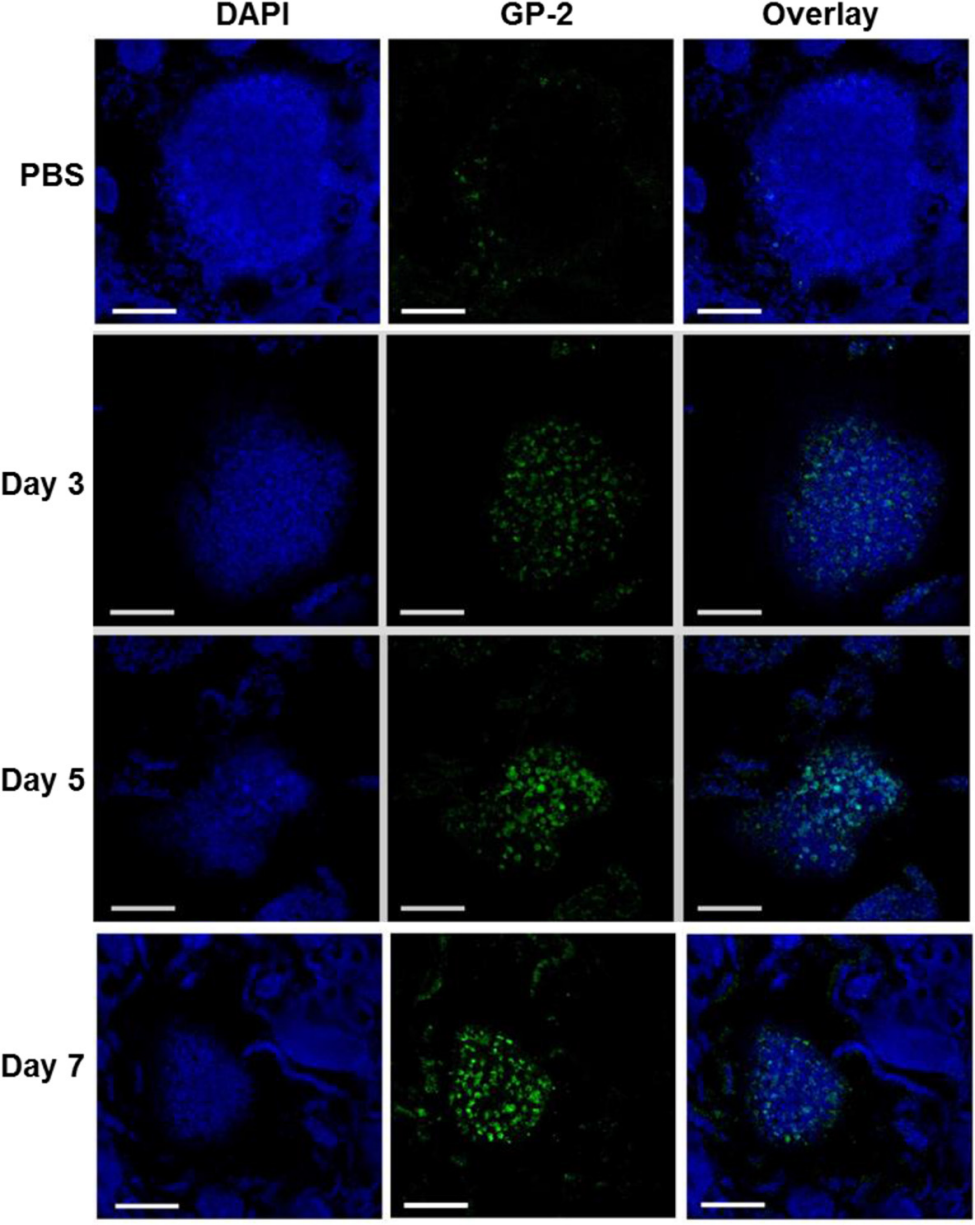
Representative images for whole mount IHC of M cells according to time. At day 3, 5 and 7 post-treatment of sRANKL-LAB, Peyer’s patches were isolated from intestine and stained with Alex 488-labeled anti-GP-2, to observe the progress of M cell development by sRANKL. The nucleus was stained with DAPI. Scale bar represents 100 μm

sRANKL-LAB was orally administered to mice for consecutive seven days based on prior studies [15], and the concentration of M cells in Peyer’s patches was analyzed by whole mount immunohistochemistry (IHC). On day 8, Peyer’s patches were isolated from the small intestine, and an M cell-specific marker GP-2 was used for immunostaining. As shown in Fig. 4a, GP-2 activity was clearly increased in the sRANKL-LAB group. The intensity of GP-2 was 1.51-fold and 1.31-fold higher than the control and WT-LAB group, when analyzed using the ImageJ program. In addition, morphometric analysis [29] indicated that the density of GP2^+^ cells was 1.68-fold and 1.54-fold higher than the control and WT-LAB group, respectively (Fig. 4b). These results suggests that secreted sRANKL from orally administered sRANKL-LAB efficiently induced M cell development in vivo. In addition, the kinetics of M cells development by sRANKL-LAB was observed by staining for GP-2 at days 3, 5 and 7 after the first treatment with sRANKL-LAB. An increase of GP-2 signal was observed on day 3, and further increases of the signal were shown on days 5 and 7 (Fig. 5).

### Evaluation of recombinant sRANKL as a potent mucosal adjuvant

**Fig. 6:**
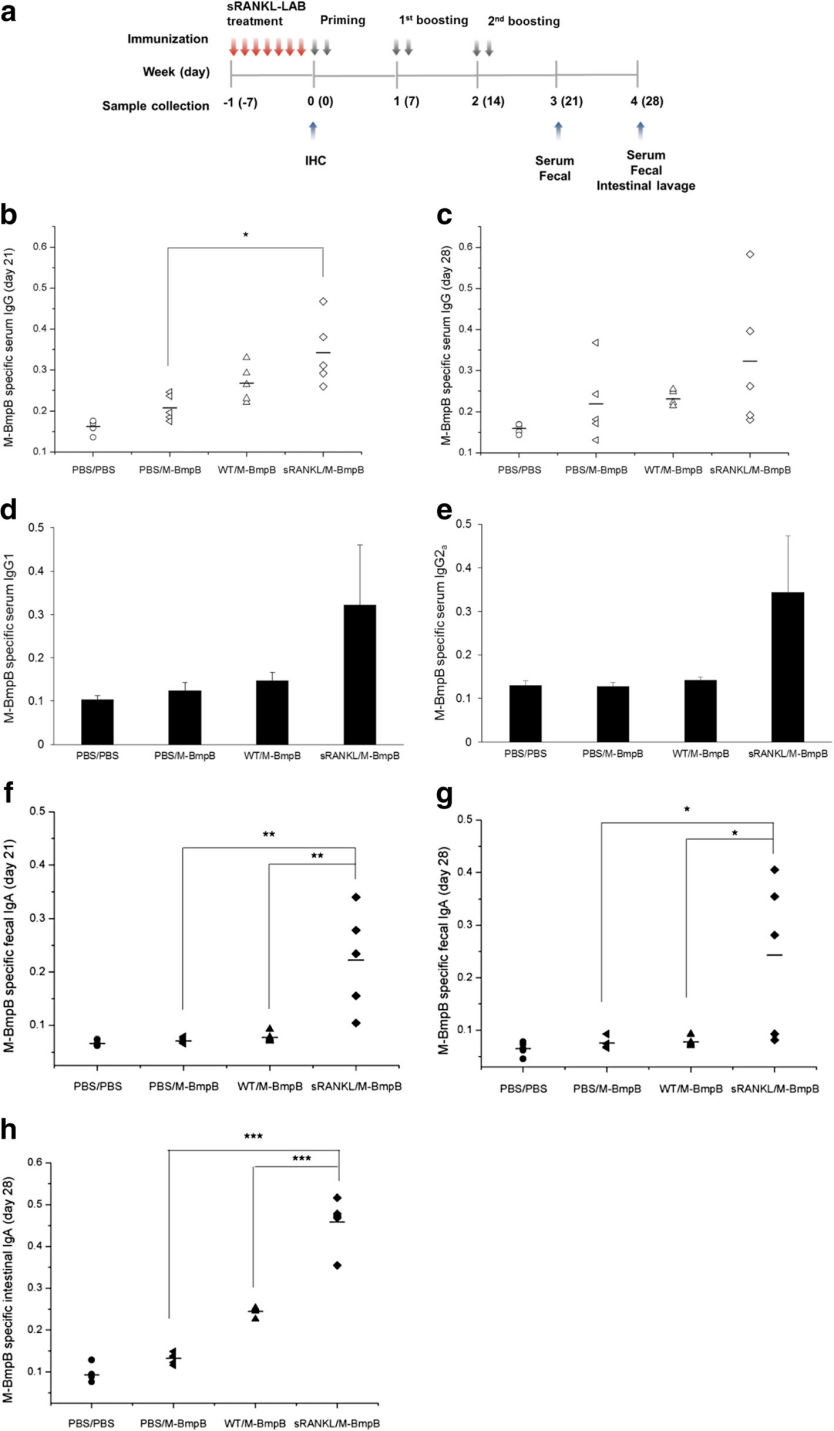
Validation of sRANKL-LAB as oral adjuvant in vivo. **a** Schematic view of sRANKL-LAB treatment, priming-boosting and sampling schedule (*n* = 5 in each group tested). **b** M-BmpB-specific total serum IgG levels at days of 21 and **c** 28, and **d** IgG1 subtype and **e** IgG2a subtype at day 28 after priming were determined by ELISA and expressed as OD value (450 nm). **f** M-BmpB-specific fecal IgA levels at days of 21 and **g** 28, and **h** intestinal IgA levels at day 28 were measured with the same method. PBS/PBS; oral administration with PBS for every 7 days and immunization with PBS. PBS/M-BmpP; oral administration with PBS for every 7 days and immunization with M-BmpB. WT/M-BmpP; oral administration with 2 × 10^8^ CFU of WT-LAB for every 7 days and immunization with M-BmpP. sRANKL/M-BmpP; oral administration with 2 × 10^8^ CFU of sRANKL-LAB every 7 days and immunization with M-BmpB. For significance tests, a one-way analysis of variance (ANOVA) followed by Tukey’s post-hoc test were used, and expressed as follows; *P < 0.05, **P < 0.01, ***P < 0.001

To investigate the adjuvant effect of sRANKL, the model antigen M-BmpB was administered orally after treatment with sRANKL-LAB. The immunization schedule and the time of sampling are shown in Fig. 6a. At days 21 and 28 after the first immunization, the levels of M-BmpB-specific total serum IgG, IgG1, IgG2a, fecal IgA and intestinal IgA were monitored by ELISA and expressed as OD values. M-BmpB specific serum IgG in the sRANKL-LAB group was 1.27-fold and 1.39-fold higher than WT-LAB at days 21 and 28, respectively (Fig. 6b and c). The level was 1.63-fold and 1.47-fold higher than the PBS-treated groups at days 21 and 28, respectively. Although significant differences in serum IgG were not observed between the IgG levels of WT-LAB and sRANKL-LAB groups, consistent enhancements of IgG in the sRANKL-LAB group were observed. To investigate the effect of sRANKL on IgG subtypes, we analyzed serum IgG1, a Th2 antibody isotype and IgG2a, a Th1 isotype. Both IgG1 and IgG2a levels were elevated by treatment with sRANKL-LAB; however, an alteration of Th1 and Th2 antibody balance was not observed (Fig. 6d and e).

We also analyzed the effect of sRANKL-LAB treatment on fecal and intestinal IgA production. Fecal IgA levels in the sRANKL-LAB-treated group were significantly higher than the WT-LAB-treated group (2.86-fold and 2.53-fold greater at 21 and 28 days, respectively) and the PBS-treated group (3.12-fold and 2.85-fold greater at day 21 and 28, respectively) (Fig. 6f and g). Similarly, at day 28 after the first immunization, oral administration of sRANKL-LAB also enhanced intestinal IgA levels (Fig. 6h). These results clearly show that M cell development by pre-treatment with sRANKL-LAB improved the induction of M-BmpB IgA and IgG antibody responses.

## Discussion

M cell induction by microbes and toxins is a protective strategy that increases the gut’s ability to transport pathogens into lymph nodes, thus initiating a protective immune response. In that context, we hypothesized that stimulation of M cells before vaccination would affect the mucosal immune response. RANKL^−/−^ mice have less than 2 % of wild-type levels of M cells in Peyer’s patches, and the M cell deficit is corrected by administration of exogenous RANKL, implying a critical role of RANK-RANKL signaling in M cell development. The effect of oral administration of sRANKL on M cells, however, has not been investigated. In this work, sRANKL was administered orally for two reasons: first, to measure its impact on the gastrointestinal tract where Peyer’s patches exist, and second, to make administration easier.

We utilized *L. lactis* as a live oral delivery vehicle by engineering it to express RANKL because *L. lactis* can survive passage through the stomach and bile acid. In general, the production of exogenous protein by LAB can occur in three different locations: intracellular, extracellular and cell wall-anchored [30]. It has been generally accepted that cell-wall anchored antigens provide the highest immune responses due to easy processing by antigen-presenting cells [30]. However, sRANKL must directly interact with RANK expressed on the subepithelium; thus, sRANKL-LAB was constructed to secret sRANKL extracellularly by conjugating it with the usp45 signal peptide, which targets proteins to the cytoplasmic membrane. Following cleavage of the usp45 peptide, the mature sRANKL protein is released extracellularly, resulting in a direct interaction with the environment. We measured a 24 % secretion efficiency (60 ng/ml) when analyzed by western blot and ELISA. In addition, strong and constitutive expression of sRANKL was driven by a Ptuf promoter, which was previously isolated by our group [26] and determined to work in vitro and in the gastrointestinal tract [3]. The high-level protein expression can overburden the metabolic capacities of the host and may inhibit normal physiology. In spite of high-efficiency sRANKL expression, the growth pattern and lactic acid production of sRANKL-LAB were similar to WT-LAB, suggesting that the basic physiology of the recombinant strain was not severely modified.

Furthermore, we characterized the functional activity of sRANKL produced from sRANKL-LAB in vitro. RANKL is a key cytokine in osteoclast differentiation via the RANK-RANKL signaling pathway. Therefore, the expression of RANK-RANKL signaling molecules is a good indicator of the function of recombinant sRANKL. We analyzed TRAF5, NFATc1 and TRAP gene expression as key molecules in the RANK-RANKL signaling pathway by real-time quantitative reverse transcription PCR (qRT-PCR). Binding of RANKL to RANK expressed on RAW 264.7 results in recruitment of TRAF6 (tumor-necrosis-factor-receptor-associated factor 6), which activates NF-kB and JNK/c-Jun, and stimulates c-Fos induction. NF-kB and c-Fos induce NFATc1 (nuclear factor of activated T cells, cytoplasmic 1) expression and in the nucleus, NFATc1 works together with other transcription factors to induce various osteoclast-specific genes including TRAP (tartrate-resistant acid phosphatase) [31, 32].

The culture media containing recombinant sRANKL and commercial sRANKL promoted the expressions of RANK-RANKL signaling molecules, while the culture media of WT-LAB did not induce the signaling pathway when analyzed by qRT-PCR. Although the expression levels of RANK-RANKL signaling-related genes were higher in the recombinant sRANKL group than in the commercial sRANKL group, a comparison of their functional activity seems inappropriate because they have different positions and expression sites. The commercial sRANKL contains amino acids 152–317, while our recombinant sRANKL contains amino acids 137–316 [15]. In addition, the presence of erythromycin in the culture medium of sRANKL-LAB may affect the expression of RANK-RANKL signaling-related genes.

This study was inspired by a report demonstrating that exogenous administration of RANKL nearly reconstitutes the M cells of RANKL^−/−^ mice, proving the M cell developmental function of RANKL even after the immune system has matured [15]. First, we examined whether M cell development could be stimulated by oral administration of sRANKL-LAB. Daily administration of 2 × 10^8^ CFU (10^9^ CFU/ml) of sRANKL-LAB for seven days led to an approximately 1.5-fold increase of M cells in Peyer’s patches. Given the Ptuf promoter activity in the intestinal tract [3], it is reasonable to assume that the secreted sRANKL elicited RANK-RANKL signaling in the subepithelium. Second, the efficacy of sRANKL-LAB as an oral adjuvant was validated by oral vaccination of mice with M-BmpB, a model antigen [24]. M-BmpB is comprised of an M cell targeting peptide isolated by phage display, and BmpB, an outer envelope protein from *Brachyspira*. M-BmpB was previously shown to target M cells and elicit higher antibody responses than a conventional BmpB antigen. Hence, it was hypothesized that an increase of M cell numbers would enhance mucosal immune responses. After induction of M cell development with sRANKL-LAB, priming-boosting with M-BmpB was conducted via the oral route. Treatment with the M-BmpB antigen without sRANKL-LAB did not elicit an antibody responses, which implies that oral vaccination with soluble protein antigens requires an efficient mucosal adjuvant. Although no significant difference was detected in M-BmpB-specific serum IgG between the sRANKL-LAB-treated group and the WT-LAB-treated group, a trend of enhancement of serum IgG responses was observed in the sRANKL-LAB group. The ratios of IgG2a to IgG1, an indicator of Th1 or Th2 type responses, respectively, were not altered by treatment with sRANKL-LAB. Importantly, IgA in fecal extracts and intestinal lavage fluid in the sRANKL-LAB group was significantly higher than the other groups, which suggests that M cell development by sRANKL increased the antigen transcytotic capability of FAE, and thereby enhanced the mucosal immune response. Our results demonstrate the potential of our adjuvant candidate, sRANKL. Although oral administration of sRANKL-LAB for seven days stimulated M cell development and enhanced mucosal immune responses, an optimal schedule of adjuvant treatment needs to be further explored to maximize vaccine effect.

## Conclusions

M cells play a central role in the initiation of an intestinal immune response by transporting foreign antigen to Peyer’s patches. In that sense, increasing the number of M cells could be a promising strategy for enhancing oral vaccine potency. Our approach was based on the knowledge that RANKL signaling is sufficient to induce the M cell development in the intestine. To utilize the RANKL as novel oral adjuvant, we constructed recombinant *L. lactis* IL1403 expressing and secreting sRANKL, and demonstrated its adjuvant potential in vivo. We found that oral administration of sRANKL-LAB enhances the systemic and mucosal immune responses to oral vaccine by increasing M cell number, and thus facilitating the transcytotic ability of FAE.

## Methods

### Microorganism strains and growth conditions

*E.coli* DH5α and *L. lactis* IL1403 were used as host strains. *E.coli* DH5α was grown in Luria-Bertani (LB) medium (Difco, BD, USA) and recombinant *E.coli* with ampicillin resistance were grown in LB medium with 100 μg/ml ampicillin at 37 °C with shaking at 250 rpm. Wild type *L. lactis* IL 1403 was grown in M17 medium (Difco, BD, USA) supplemented with 5 g/L of glucose (M17G) without antibiotics, and recombinant *L. lactis* was grown in M17G media with 5 μg/ml of erythromycin at 30 °C with shaking.

### Plasmid construction and transformation of *L. lactis*

To construct recombinant sRANKL-LAB, the pILPtuf vector system previously constructed by our group was used as a backbone [26]. The 181 amino acid sequence of soluble RANKL was obtained from the literature [15] and the codon usage was optimized for expression by *L. lactis*. The gene encoding a Usp45 signal peptide was cloned upstream of the sRANKL gene to induce secretion of the protein. The *Nde*1-Usp45-sRANKL-*Xho*1 gene fragment cloned into the pGEM-B1 vector by Bioneer (South Korea). To insert the Usp45-sRANKL gene fragment into the pILPtuf vector, pGEM-B1 was digested by *Ned*1 and *Xho*1 restriction enzymes, and introduced into the pIL252 backbone using the same restriction sites and T4 DNA Ligase (NEB, USA). A schematic illustration of the expression constructs is shown in Fig. 1. After pellet paint coprecipitation to remove salts from the ligation products, the vector was transformed into *L. lactis* IL 1403 by electroporation (2.5 kV, 10 μF, and 300 Ω). The transformant candidates were confirmed by DNA sequencing at the National Instrumental Center for Environmental Management (NICEM, South Korea).

### Confirmation of the expression and secretion of sRANKL from LAB

The expression and secretion of sRANKL from sRANKL-LAB was measured by SDS-PAGE and western blot. Commercial sRANKL (amino acids 152–317 of RANKL) obtained from Enzo Life Science (USA) was used as a control protein. Transformed *L. lactis* was cultured in M17G media supplemented with erythromycin at 30 °C for overnight, and the intracellular and secreted proteins were harvested separately. For preparation of intracellular protein, cells were collected by centrifugation at 13,000 rpm for 1 min and broken in a bead beater. The secreted proteins were precipitated from *L. lactis* culture supernatants by 16 % trichloroacetic acid (TCA), and the precipitants were washed with ethanol and dissolved in Tris–HCl buffer.

The sample proteins separated by SDS-PAGE were transferred onto a 0.2 μm nitrocellulose membrane (Whatman, USA). The membrane was blocked with 5 % (w/v) skim milk/TBST at RT for 1 h with shaking, and subsequently incubated with a goat anti-mouse RANKL antibody (R&D Systems, USA) at a dilution of 1:000 in TBST at 4 °C overnight with shaking. The membrane was further washed in TBST and incubated with anti-goat antibody (Santa Cruz Biotechnology, USA) (1:1000 dilution) at room temperature (RT) for 1 h. The immunoreactive proteins were visualized with ECL reagents (Amersham, USA) using a ChemiDoc system (Bio-Rad, USA). sRANKL expression was quantified using a mouse RANKL solid sandwich ELISA kit (R&D Systems, USA) according to the manufacturer’s instructions. In brief, assay diluent was added to each well coated with RANKL antibody, and a serial dilution of intracellular or extracellular protein sample prepared by above-mentioned method, or calibrator RANKL protein was added to each well. After incubation for 2 h and washing step, enzyme-conjugated antibodies were applied as detection antibodies, and substrate solution was added each to convert to colorimetric detectable form. After measuring OD value of each well at 450 nm wavelength, dilutions of protein samples within detectable range were quantified.

### Physiological characterization of sRANKL-LAB

sRANKL-LAB and WT-LAB were cultured in 50 ml of M17G broth supplemented with erythromycin and M17G broth without antibiotics, respectively. The optical density (OD) of each sample was measured at a wavelength of 600 nm every 2 h for 12 h and at 24 h after inoculation to determine cell growth. The pH value of the LAB cultures was measured simultaneously.

### Validation of the biological activity of recombinant sRANKL in vitro

RAW 264.7 cells obtained from the Korea Cell Line Bank (KCLB) were seeded on cell culture dishes in DMEM (Hyclone, USA) with 10 % fetal bovine serum (FBS) (Hyclone, USA) and 1 % penicillin at a density of 2 × 10^4^/ml. At four hours post seeding, the media was changed to DMEM with 10 % FBS, 1 % P/S, and 30 ng/ml of M-CSF with or without 20 ng/ml of sRANKL.

The recombinant sRANKL from the supernatant of sRANKL-LAB cultures was prepared in a crude form as described above and applied to RAW 264.7 cells at a concentration of 20 ng/ml. The supernatant of WT-LAB cultures and commercial sRANKL (20 ng/ml) were also applied to RAW 264.7 cells for positive and negative controls. At three days post treatment, the media was replaced by fresh media and the cells were incubated a further three days. Total RNA was isolated from the RAW264.7 cells using TRizol according to the manufacturer’s protocol and cDNA was synthesized using an AccuPower® CycleScript RT PreMix (oligo dT) (Bioneer, South Korea). qRT-PCR was conducted using TOPreal qPCR 2X PreMIX (Enzynomics, South Korea) on iCycler Real-Time Detection System (BioRad, USA) with specific primers as follows: NFATc1-F 5′-TCCTGCTCCTCCTCCTGCTGCTCG-3′; NFATc1-R 5′-GCTGCTGGCAAGGCAGAGTGTGCT-3′; TRAF6-F 5′-GCCTGCATCATCAAATCCATAAGG-3′; TRAF6-R 5′-AATTCACAATGTACTTGATGATCCTCG-3′; TRAP-F 5′-GCGACCATTGTTAGCCACATACGG-3′; TRAP-R 5′-CGCCCAGGGAGTCCTCAGATCCAT-3′; GAPDH-F 5′-AACTTTGGCATTGTGGAAGGGCTC-3′; GAPDH-R 5′-AAGGCCATGCCAGTGAGCTTC-3′. For quality assurance, melting curve analysis was performed after completing the qRT-PCR. The amplicons from NFATc1, TRAF6, TRAP and GAPDH each produced a single peak, demonstrating reaction specificity and the absence of primer-dimer artifacts.

### Validation of the biological activity of recombinant sRANKL in vivo

The effect of sRANKL-LAB on M cell development was measured in vivo using six-week-old BALB/C mice following the policy and regulations for the care and use of laboratory animal (Laboratory Animal Center, Seoul National University, Korea). All protocols were reviewed and approved by the Animal Care and Use Committee at Seoul National University (SNU-130506-3). sRANKL-LAB and WT-LAB for in vivo experiments were grown in M17G broth at 30 °C overnight, and cell pellets were harvested by centrifugation when the OD_600nm_ reached 2.0. Cells were resuspended in 1 ml M17G broth at a concentration of 10^9^ CFU/ml and further incubated at 30 °C for 1 h. Thirty min before LAB administration, each mouse was gavaged with 500 μl of a neutralizing agent (1.5 % NaH_2_CO_3_) to prevent gastric acidity. LABs or PBS (200 μl) were orally administered using an oral Zonde needle for seven consecutive days (*n* = 5 mice/group and time point).

One day after completing seven days of sRANKL-LAB treatment, mice were killed and the Peyer’s patches of the small intestine were collected for IHC. The detached Peyer’s patches were incubated with PBST supplemented with DNase (500 unit/ml) at 37 °C for 20 min, followed by washing with cold PBST three times and fixation with 4 % (v/v) paraformaldehyde for 2 h at 4 °C. After washing three times, the tissue was blocked with 3 % goat serum at RT for 1 h, and incubated with an Alexa488-labeled GP-2 monoclonal antibody (1:400 dilution) at 4 °C overnight. The nucleus was stained with 1 mg/ml of 4′, 6-Diamidine-2-phenylindole (DAPI) and the tissue was mounted on cover-glass bottom dishes. The fluorescence signals were observed by a Confocal Laser Scanning Microscope (SP8X STED, Leica, Germany). The relative GP-2 expression and GP-2^+^ cell density were evaluated using Image J software according to the method described in [29]. A schematic illustration of the experimental steps of IHC is provided in Additional file 1: Figure S2.

### Immunization of mice in vivo

One day after treatment with sRANKL-LAB for seven consecutive days (day 0), mice were orally immunized with M-BmpB (*n* = 5 mice/group), prepared as described in [3], using the immunization schedule as shown in Fig. 2. The immunization groups were as follows: (i) 200 μl of PBS after treatment with PBS for seven days (ii) 200 μg of M-BmpB after treatment with PBS for seven days (iii) 200 μg of M-BmpB after treatment with WT-LAB for seven days (iv) 200 μg of M-BmpB after treatment with sRANKL-LAB for seven days. M-BmpB, a model antigen [24], was prepared by culturing recombinant *E.coli* expressing a pET-M-BmpB plasmid, and purifying M-BmpB using nickel affinity chromatography (BioRad, USA). Thirty min before M-BmpB treatment, each mouse was gavaged with a neutralizing agent as described above, and subsequently given M-BmpB dissolved in 200 μl of PBS using an oral Zonde needle.

Blood and fecal samples were collected on days 21 and 28 after primary immunization. Blood samples were collected from the intra-petrosal veins with a disposable syringe and delivered into a sterilized tube. Serum was separated by centrifugation at 12,000 rpm for 10 min, and then used for detecting M-BmpB-specific immunoglobulins. For fecal sample preparation, 5–8 fecal pellets were collected and suspended in PBS. After intensive vortexing, fecal samples were incubated at 4 °C overnight, and the supernatants were collected by centrifugation at 14,000 rpm for 10 min. Intestinal lavage samples were collected on day 28 after primary immunization. Mice were orally administered 0.5 ml of the lavage solution (25 mM NaCl, 40 mM Na_2_SO_4_, 10 mM KCl, 20 mM NaHCO_3_, and 48.5 mM polyethylene glycol (MW 3350)) four times at intervals of 15 min using a blunt Zonde. At 30 min post-administration, 0.1 mg pilocarpine was given to the mice intraperitoneally, and the discharged intestinal contents (up to 0.5 ml) were collected for measuring M-BmpB-specific IgA.

### ELISA assay

The M-BmpB-specific immunoglobulins from serum or fecal samples were measured by ELISA as previously described [3]. In brief, purified M-BmpB was coated onto 96 well immuno-plates, and 100 μl of diluted serum or fecal samples were loaded and incubated at RT for 2 h. Diluted HRP-conjugated goat anti-mouse IgG, IgG1, IgG2a or IgA (Santa Cruz Biotechnology, USA) was added to each designated well at 37 °C for 1 h. TMB substrate buffer (Santa Cruz Biotechnology, USA) was added to each well, and stopped after 10 min by treating with 100 μl of stop solution. The ELISA results are expressed as the OD values measured at 450 nm by a microplate reader (Infinite M200, TECAN, Germany) using dilutions of 1:100 for serum, 1:2 for fecal extracts and 1:3 for intestinal samples.

### Statistical analysis

Statistical analysis was performed using OriginPro 9.0 software (OriginLab, USA). For significance tests, a one-way analysis of variance (ANOVA) followed by Tukey’s post-hoc test were used, and expressed as follows; *P < 0.05, **P < 0.01, ***P < 0.001.

## Additional file

Additional fie 1:
**Figure S1.** qRT-PCR analysis of RANK-RANKL signaling-related gene expression to optimize the concentration of commercial sRANKL. The mRNA expressions of TRAF6, NFATc1, and TRAP were analyzed at day 6 after exposure of commercial sRANKL (20-50 ng/ml) to RAW 264.7 cells. The mRNA levels were normalized by GAPDH expression, and expressed as relative gene expression compared to control. For significance tests, a one-way analysis of variance (ANOVA) followed by Tukey’s post-hoc test were used, and expressed as follows; **P* < 0.05, ***P* < 0.01, ****P* < 0.001. **Figure S2.** Schematic illustration of IHC. Peyer’s patches were isolated from small intestine and fixed with 4 % (v/v) paraformaldehyde for 2 h at 4 °C. The tissues were blocked with 3 % goat serum at RT for 1 h and incubated with Alexa488-labeled GP-2 monoclonal antibody (1:400 dilution) at 4 °C overnight. The tissues were mounted on coverglass bottom dishes. (PDF 232 kb)

## Abbreviations

FAE: Follicle-associated epithelium
GALT: Gut-associated lymphoid tissue
qRT-PCR: Real-time quantitative reverse transcription PCR
IHC: Immunohistochemistry
*L. lactis*: *Lactococcus lactis*
RANKL: Receptor activator of NF-kB ligand
sRANKL: Soluble receptor activator of NF-kB ligand
sRANKL-LAB: recombinant *L. lactis* 1403 secreting sRANKL
WT-LAB: Wild-type *L. lactis* IL1403
M-BmpB: *Brachyspira* membrane protein B conjugated with CKS9
