# Supplementary material for: Soluble RANKL expression in Lactococcus lactis and investigation of its potential as an oral vaccine adjuvant
Source: BMC Immunol. 2015 Nov 25;16:71. doi: 10.1186/s12865-015-0132-x (PMC4659156; doi:10.1186/s12865-015-0132-x)
Supplement: Additional fie 1: — Figure S1. qRT-PCR analysis of RANK-RANKL signaling-related gene expression to optimize the concentration of commercial sRANKL. The mRNA expressions of TRAF6, NFATc1, and TRAP were analyzed at day 6 after exposure of commercial sRANKL (20-50 ng/ml) to RAW 264.7 cells. The mRNA levels were normalized by GAPDH expression, and expressed as relative gene expression compared to control. For significance tests, a one-way analysis of variance (ANOVA) followed by Tukey’s post-hoc test were used, and expressed as follows; *P < 0.05, **P < 0.01, ***P < 0.001. Figure S2. Schematic illustration of IHC. Peyer’s patches were isolated from small intestine and fixed with 4 % (v/v) paraformaldehyde for 2 h at 4 °C. The tissues were blocked with 3 % goat serum at RT for 1 h and incubated with Alexa488-labeled GP-2 monoclonal antibody (1:400 dilution) at 4 °C overnight. The tissues were mounted on coverglass bottom dishes. (PDF 232 kb) [file 12865_2015_132_MOESM1_ESM.pdf]

Supplementary information

Soluble RANKL expression in *Lactococcus lactis* and investigation for its potential as an oral vaccine adjuvant

Jeong-In Kim[1,#], Tae-Eun Park[1,#], Sushila Maharjan[1], Hui-Shan Li[1], Ho-Bin Lee[1], In-Seon Kim[1], Dachuan Piao[1], Jun-Yeong Lee[1], Chong-Su Cho[1], Jin-Duck Bok[2], Zhong-Shan Hong[3], Sang-Kee Kang[2*], Yun-Jaie Choi[1,3*]

[1]Department of Agricultural Biotechnology & Research Institute for Agriculture and Life Sciences, Seoul National University, Seoul, South Korea; [2]Institute of Green-Bio Science & Technology, Seoul National University, Pyeongchanggun, Gangwondo, South Korea; [3]Department of Animal Science, Tianjin Agricultural University, Tianjin, China[#] Jeong-In Kim and Tae-Eun Park contributed equally to this work

Sang-Kee Kang; Tel.: +82-33-339-5731; Fax : +82-33-339-5732; E-mail : kangsk01@snu.ac.kr

Yun-Jaie Choi; Tel.: +82-2-880-4807; Fax : +82-2-875-7340; E-mail : cyjcow@snu.ac.kr

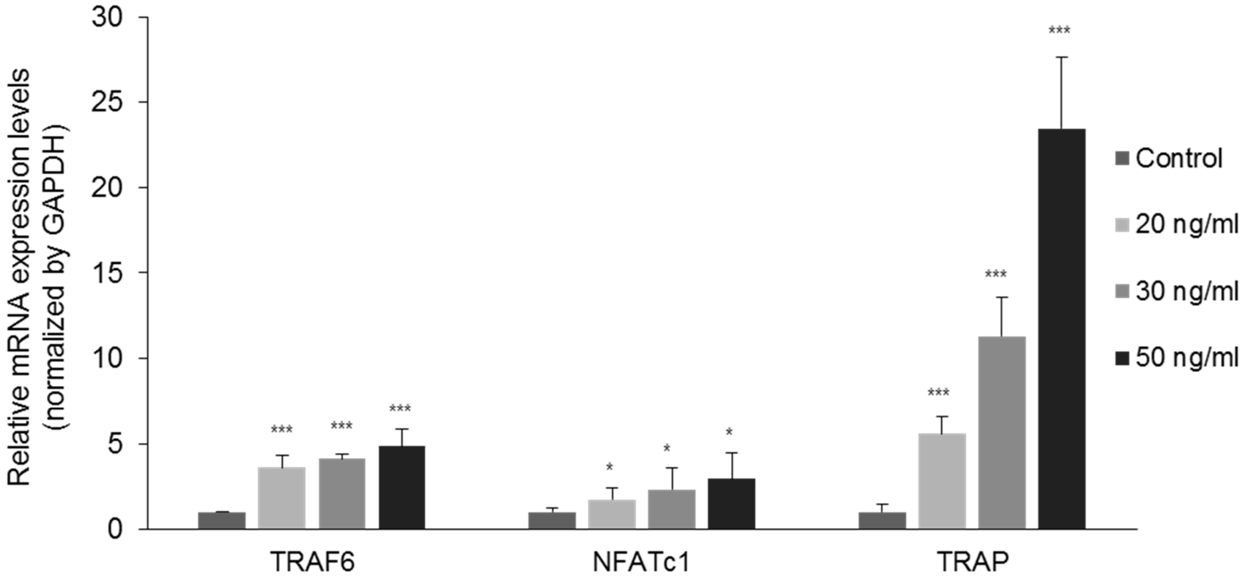

Figure S1. qRT-PCR analysis of RANK-RANKL signaling-related gene expression to optimize the concentration of commercial sRANKL. The mRNA expressions of TRAF6, NFATc1, and TRAP were analyzed at day 6 after exposure of commercial sRANKL (20-50 ng/ml) to RAW 264.7 cells. The mRNA levels were normalized by GAPDH expression, and expressed as relative gene expression compared to control. For significance tests, a one-way analysis of variance (ANOVA) followed by Tukey′s post-hoc test were used, and expressed as follows; *P < 0.05, **P < 0.01, ***P < 0.001.

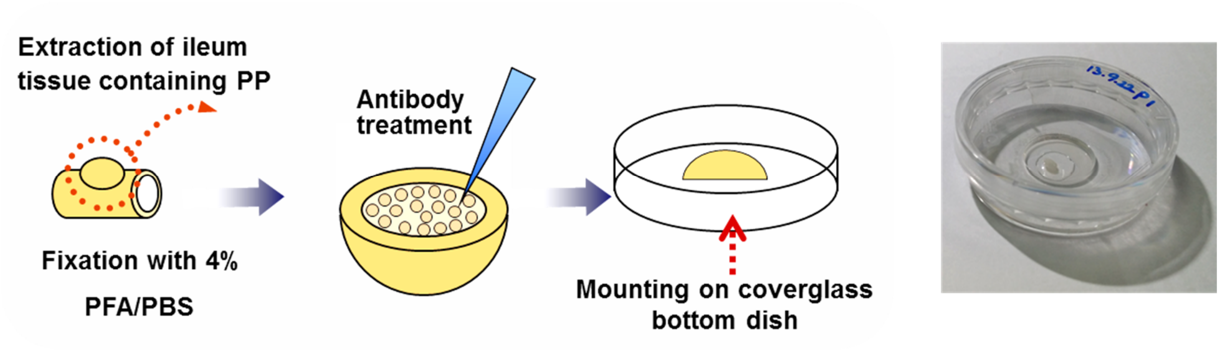

Figure S2. Schematic illustration of IHC. Peyer's patches were isolated from small intestine and fixed with 4% (v/v) paraformaldehyde for 2 h at 4°C. The tissues were blocked with 3% goat serum at RT for 1 h and incubated with Alexa488-labeled GP-2 monoclonal antibody (1:400 dilution) at 4°C overnight. The tissues were mounted on coverglass bottom dishes.
